# Pharmacological and Therapeutic Potential of Myristicin: A Literature Review

**DOI:** 10.3390/molecules26195914

**Published:** 2021-09-29

**Authors:** Elisa Frederico Seneme, Daiane Carla dos Santos, Evelyn Marcela Rodrigues Silva, Yollanda Edwirges Moreira Franco, Giovanna Barbarini Longato

**Affiliations:** 1 Research Laboratory in Molecular Pharmacology of Bioactive Compounds, São Francisco University (USF), Bragança Paulista 12916900, SP, Brazil; elisaseneme@gmail.com (E.F.S.); daiiics93@gmail.com (D.C.d.S.); evelynmrsilva@gmail.com (E.M.R.S.); 2 Graduate Program in Health Science, São Francisco University, Bragança Paulista 12916900, SP, Brazil; yollanda.moreiraf@gmail.com; 3 Laboratory of Molecular and Cellular Biology (LIM), Department of Neurology, Faculdade de Medicina FMUSP, Universidade de São Paulo, São Paulo 01246903, SP, Brazil

**Keywords:** myristicin, nutmeg, natural products, bioactive compounds, therapeutic properties

## Abstract

Natural products have been used by humanity for many centuries to treat various illnesses and with the advancement of technology, it became possible to isolate the substances responsible for the beneficial effects of these products, as well as to understand their mechanisms. In this context, myristicin, a substance of natural origin, has shown several promising activities in a large number of in vitro and in vivo studies carried out. This molecule is found in plants such as nutmeg, parsley, carrots, peppers, and several species endemic to the Asian continent. The purpose of this review article is to discuss data published in the last 10 years at Pubmed, Lilacs and Scielo databases, reporting beneficial effects, toxicity and promising data of myristicin for its future use in medicine. From 94 articles found in the literature, 68 were included. Exclusion criteria took into account articles whose tested extracts did not have myristicin as one of the major compounds.

## 1. Introduction

Biodiversity is the variability of all living beings in the biosphere, in its entirety. Such beings become renewable sources of substances that can originate various products for human consumption, including medicines for the treatment of various pathologies. The main producers of these substances are plants, microorganisms, marine algae, among others, which over thousands of years of evolution, were capable of adaptations that made them capable of acquiring biological activities of various types [1].

Data show that there are still few drug discovery programs based on natural products in pharmaceutical companies, although they are a promising source of new drugs [2]. Even so, drugs produced from natural substances are numerous, since those obtained from natural sources represent about 70% of all drugs approved for therapeutic use in the last four decades [3].

Natural compounds have been one of the main sources of the production of medicines since the beginning of time, giving rise to drugs of different therapeutic classes. One of the main examples is the discovery of penicillin in 1928, by the researcher Alexander Fleming, whose research with fungi of the *Penicillium* genus culminated in the discovery of a compound with an antibacterial activity. This compound was called penicillin and is currently used to treat infections caused by bacteria of the *Staphylococcus genus* [4]. Another natural compound of great importance is quinine, derived from the bark of the quinine. Initially this herb was consumed by the indigenous people of the Amazon region. This plant has been used for decades to treat malaria, and this substance gave rise to other drugs to treat the disease, such as chloroquine [2]. *Arnica montana* plant species, also widely used in Brazil for many years, has anti-inflammatory, analgesic and healing actions that improve or prevent injuries, and currently its extract can be found in ointments and gels produced by the pharmaceutical industry [5]. Therefore, since the main source of new medications are natural products, it is necessary to carry out research to discover new treatments from sources that have been little explored. 

In this work, we will discuss a substance called myristicin. It was first discovered in the seed of nutmeg (*Myristica fragrans*), and was described in the French colonies in the mid-18th century, on the Maluku islands [6]. In addition to the high concentration in this seed, myristicin can also be found in cinnamon, parsley, some types of pepper and other spices native to Asia. Nutmeg was used in ancient times (in India and other regions of Asia) to treat anxiety, stomach cramps, nausea and diarrhea [7]. In addition, it has been described as a food preservative, as it has antimicrobial activities, and it is currently used as a flavoring agent by the food industry [8]. When used in very high amounts, myristicin can have toxic effects, leading to liver degeneration and mental confusion, as it is toxic to the central nervous system. It is believed that myristicin is in the main responsible for the benefits described with the use of nutmeg, as well as for its toxic effects, since it is the largest compound present in this spice [7].

Several preliminary studies have been conducted with myristicin over the last few years, demonstrating that it has promising biological activities, but it is still little explored. Thus, considering the ethnopharmacology of myristicin, as well as the importance of natural products as a source of new drugs, there is an urgent need to investigate scientific data about its properties, which may justify its use as a therapeutic substance in addition to arousing scientific interest in continuing the investigation of its pharmacological properties.

## 2. Results and Discussion

### 2.1. Metabolization and Toxicity of Myristicin

**Figure 1:**
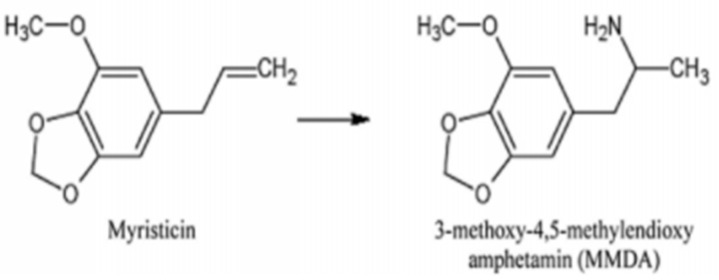
Molecular structure of myristicin and its active metabolite MMDA [9].

In the 1960s and 1970s, nutmeg was used as a psychedelic drug by the hippie culture, but it was abandoned due to the headache it caused in users. The main toxic activity of nuts occurs in the central nervous system, and is directly linked to the high concentrations of myristicin (1-allyl-5-methoxy-3,4-methylenedioxybenzene), although there may be synergistic effects with the other components [9,10]. The psychedelic effects of myristicin are thought to be related to its active amphetamine-derived metabolite. Furthermore, myristicin is slightly capable of inhibiting the enzyme monoamine oxidase (MAO), which would cause pro-serotoninergic effects and cardiovascular symptoms. Studies have shown that myristicin is able to promote anxiogenesis and affect motor actions and it is suggested that it is able to modulate GABA receptors, possibly acting as an antagonist, generating anxiety [11,12,13,14]. Myristicin is metabolized in the liver by enzymes of the cytochrome P450 complex. Its hepatic biotransformation generates metabolites that remain active and may be responsible for its toxicity. In phase 1 metabolism, the main active metabolites are 1’-hydroxymyristicin and 5-allyl-1-methoxy-2,3-dihydroxybenzene. It has also been reported that myristicin can be converted to an amphetamine-like metabolite: 3-methoxy-4,5-methylenedioxy-amphetamine (MMDA), known for its psychedelic effects (Figure 1). The main enzyme responsible for its bioactivation is CYP1A1. Therefore, inducing substances of the CYP1A group may facilitate the formation of reactive metabolites and increase the risk of toxicity related to myristicin. However, myristicin was also evaluated for its likely ability to inhibit CYP complex enzymes, and the results showed that myristicin exerts some inhibitory effect on human CYP2E1, CYP2C19, and CYP1A2 but exerts the strongest inhibitory effect on CYP1A2 [9,10,13].

After phase 2 metabolism, myristicin metabolites are able to form complexes with endogenous N-acetylcysteine, forming the myristicin-NAC complex, as well as glutathione, forming the myristicin-GSH complex. These complexes are mostly eliminated in urine [9,10].

The toxic effects of myristicin are dose-dependent. Neuropsychological symptoms usually develop after consuming 10 to 15 g of nutmeg, or about 400 mg of myristicin (corresponding to 6–7 mg/kg). Anticholinergic effects occur after ingestion of 25 to 28 g of nutmeg. Symptoms may appear 3 to 6 h after administration and persist for up to 72 h. The main symptoms reported are: dry mouth, facial flushing, blurred vision, hypertension, tachycardia, psychomotor agitation and restlessness; in more severe cases, patients develop delusions, dissociative episodes and visual, auditory or tactile hallucinations [11,13,14].

Although potentially serious, cases of accidental poisoning are relatively rare, as it is necessary to ingest a large amount of nutmeg to reach a plasma concentration of myristicin capable of promoting such effects. Only one fatal case of poisoning was reported for a boy, aged 8, who ate two nutmegs and passed into a comatose condition [15]. In a case study, a 29-year-old male patient who had ingested about 3 to 4 whole walnuts (18 to 28 g) presented unmotivated laughter, disorganized speech and psychomotor agitation, evolving to remission in the following days. Another case study describes the intoxication of a normally developing 3-month-old infant who presented repeated episodes of afebrile status epilepticus after ingestion of nutmeg. Phenytoin, phenobarbital and midazolam were administered to control the seizures, and the patient progressed to improvement, with no new epileptic episodes [11,14]. 

From these data, it is possible to observe that myristicin and its active metabolites have psychoactive activity, which is mainly responsible for its toxicity. However, due to this activity, it can also be explored as a potential therapeutic agent to treat central nervous system pathologies [11,14].

### 2.2. Antioxidant Activity

Investigation studies of antioxidant activity were conducted with myristicin isolated or present in extracts and essential oils obtained from the following plant species: *Dorema glabrum* (14% myristicin); *Naturtium officinale* (57% myristicin); *Athamanta turbith* (52% myristicin), *Heracleum transcaucasicum* and *Heracleum anisactis* (containing 96.87% and 95.15% myristicin respectively) and *Myristica fragrans* (containing 8% to 11% myristicin) [16,17,18,19,20,21,22].

**Figure 2:**
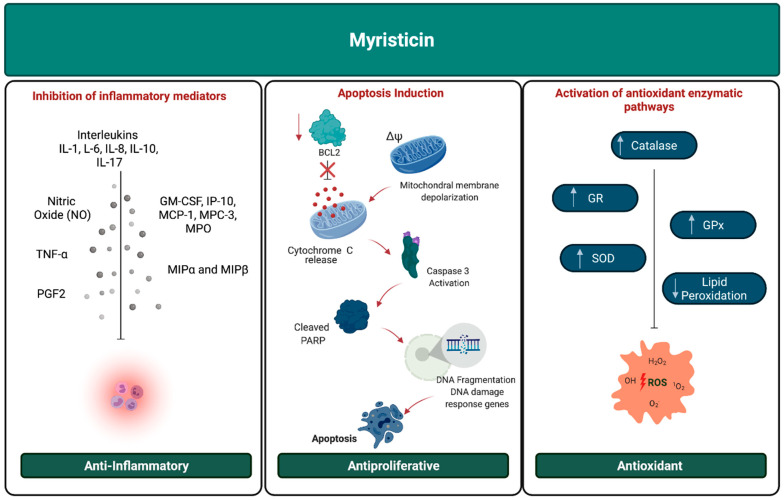
Graphical representation of the anti-inflammatory, antiproliferative and antioxidant pathway mechanisms induced by myristicin.

The results obtained showed that, in vivo, isolated myristicin (high purity) was able to increase the concentration and activity of antioxidant enzymes: catalase, superoxide dismutase, glutathione peroxidase and glutathione reductase, as well as decreasing the levels of lipid peroxidation (Figure 2). The essential oils containing myristicin also showed good antioxidant activity in vitro, with the exception of the oil from the plant *Athamanta turbith*, whose activity was relatively low [16,17,18,19,20,21,22].

One of the studies used *Myristica fragrans* (nutmeg) essential oil containing myristicin, the same oil without myristicin, and also myristicin alone. In addition to the antioxidant activity, this study provided information about a possible sun protection factor, through tests of light absorption and reactions with free radicals in vitro. The results showed that the essential oil without myristicin had minimal protection and antioxidant activity and the oil containing myristicin had moderate activity. Isolated myristicin, on the other hand, had the highest protective and antioxidant activity, which indicates that it is the substance responsible for this biological effect in nutmeg oil [23].

However, few studies indicate that myristicin is a promising antioxidant substance, which justifies further tests to understand its mechanism of action.

### 2.3. Anti-Inflammatory and Analgesic Activity

The anti-inflammatory potential of myristicin has been extensively studied in recent years. Tests to investigate this property were developed in vitro and in vivo and were conducted for isolated myristicin, as well as oils and plant extracts containing the substance in different concentrations. The main plant species used in such studies were *Trachydium roylei*, *Cinnamomum syntoc*, *Pycnocycla bashagardiana*, *Perilla frutescens*, *Myristica fragrans* (nutmeg), *Illicium lanceolatum*, *Piper chaba*, *Piper sarmentosum*, *Piper interruptum* (peppers), *Plumbago indica* and *Zingiber officinale* (ginger). In general, the tests show that myristicin is a potent anti-inflammatory. Several studies report that it is able to inhibit the production of prostaglandins (PGE2), one of the main substances involved in the inflammatory process. This activity was studied in vitro, using techniques such as Western Blotting, PCR and ELISA, and also in vivo, using paw and ear edema reduction assays in mice. Furthermore, a molecular docking study revealed that myristicin would be able to non-selectively inhibit the cyclooxygenase 2 (COX-2) enzyme, which is one of those responsible for the production of prostaglandins. However, it did not show this activity at mRNA and protein levels when treated in human liver cancer cells [24,25,26,27,28].

The anti-inflammatory activity of myristicin can also occur through other pathways (Figure 2). This molecule is also capable of inhibiting several cytokines and mediators responsible for the chemotaxis of the inflammatory process, such as: tumor necrosis factor alpha (TNF-a), interleukins (IL-1, IL-6, IL-8, IL-10 and IL-17), nitric oxide (NO), macrophage inflammatory proteins (MIP-1α r MIP-1β), colony stimulating factor (GM-CSF), IP-10, MCP-1 and MCP-3 and myeloperoxidase (MPO). This inhibition occurs both at the protein level and at the mRNA regulation level. In vitro studies have shown that the inhibition of these cytokines was able to block the migration and growth of neutrophils and macrophages, while in vivo, it promoted a reduction in mice paw edema [16,24,29,30,31,32,33,34].

The analgesic action of myristicin has also been evaluated. Tests conducted with *Pycnocycla bashagardiana* essential oil containing myristicin did not result in analgesic activity in hot plate tests with mice, despite its good anti-inflammatory action (reduction of paw edema). The essential oil of *Illicium lanceolatum*, in addition to its anti-inflammatory activity in vivo (reduction of ear edema), also showed reduced writhing in mice after pain induction by acetic acid, indicating a possible analgesic action. In this case, however, the author attributes the activity to the association between myristicin and other components of the essential oil [29,33].

Although many results were obtained through tests with essential oils containing other substances that can contribute to the anti-inflammatory action, myristicin was the major component in most of them. From these results, its anti-inflammatory activity in several pathways of the inflammation process is remarkable.

### 2.4. Antiproliferative Activity

The antiproliferative activity of myristicin has been studied in recent years. Literature data report that myristicin is responsible for the anticancer activity of some medicinal plants and is a cancer chemopreventive agent [35,36,37,38]. 

*Athamanta sicula* crude extract and isolated myristicin were tested in vitro for their antiproliferative activity, at a concentration of 100 μg/mL, against K-562 (human chronic myeloid leukemia), NCI-H460 (human non-small cell lung adenocarcinoma) and MCF-7 (human breast adenocarcinoma) cells using the methyltetrazolium (MTT) assay. The extracts and isolated myristicin showed significant antiproliferative activity in the tested cancer cell lines, with inhibition of 50% to 100% of cells at different concentrations. Other assays were used to investigate the mechanisms of growth inhibition, and it was concluded that myristicin induced cell apoptosis through changes in mitochondrial membrane potential, cytochrome C release, caspase-3 activation, PARP cleavage and fragmentation of DNA. Gene expression profiling revealed a general down-regulation of DNA damage response genes after exposure to myristicin [35,38].

Exposure of the KB cell line (human oral epidermal carcinoma) with a variable concentration of *Myristica fragrans* extract (nutmeg) resulted in a concentration-dependent inhibition of cell proliferation, suggesting that the nutmeg extract inhibited the proliferation of KB cells. The extract was able to reduce the expression of the bcl-2 gene in cells, diminishing the expression of this protein and inducing early and late apoptosis. Furthermore, the cells shrank and showed morphological changes when analyzed under a microscope. Cancer cells, however, exhibit resistance to apoptosis in order to sustain their uncontrolled proliferation, and therefore any compound that modulates apoptosis is desirable as a plausible cancer chemotherapy agent [37].

Pure and partially purified myristicin obtained from *Myristica fragrans* were tested against human rhabdomyosarcoma (RD) cells in vitro. At lower concentrations and in the first 24 h of treatment, cell growth inhibition had a significant difference: the partially purified extract showed a greater inhibitory activity. However, after 48 h of treatment and at concentrations above 125 µg/mL, both extracts showed a similar inhibitory activity. The highest rate of inhibition was 82.3%, reported at the concentration of 500 μg/mL of pure myristicin. Therefore, it is suggested that the extraction method may interfere with the biological effect; however, myristicin showed cytotoxic/antiproliferative activity for the studied strain [39]. 

The essential oil of *Myristica fragrans* containing 32% myristicin was able to induce a significant reduction in human colorectal adenocarcinoma cells (Caco-2) cell viability at the concentration of 250 μg/mL. Furthermore, myristicin isolated from the oil showed an IC50 value of 146 μg/mL, indicating that it could be the substance responsible for the cytotoxic activity of the oil [36].

Pure myristicin is also capable of inhibiting the growth of AA8 and EM9 ovarian cells. Cell viability assays were performed after treatment with different concentrations of myristicin (from 50 to 2000 μM) for 24 h, using the MTT assay protocol. The results showed a reduction in viability. Other assays were carried out, and the results showed that myristicin induced cell apoptosis through the activation of caspases (as already reported by other authors) in both strains, but mainly in EM9. However, it was not able to induce DNA damage [40].

One of the in vitro studies compared the cytotoxicity of myristicin and its active metabolite, 1’-hydroxymyristicin, against HepG2 cells, a human hepatocellular carcinoma line. Cells exposed to myristicin for 24 h did not show a significant reduction in cell viability. In contrast, cells exposed to 1’-hydroxymyristicin, in the same concentration range, showed a dramatic reduction in viability in the MTT test. A significant increase in the number of apoptotic cells (both in the early and late stages of apoptosis) was observed in cells exposed to 1’-hydroxymyristicin. These results indicate that the active metabolite of myristicin is possibly more cytotoxic and apoptotic than the substance itself [41].

Benjakul extract, a traditional medicine composed of extracts of *Piper chaba, Piper sarmentosum*, *Piper interruptum*, *Plumbago indica* and *Zingiber officinale*, which contains 3.5 mg/g of myristicin, was tested for its antiproliferative activity against human small cell lung cancer (NCI-H1688) and non-tumor human lung fibroblast cell line (MRC-5). In vitro assays have shown that benjakul is selective and can kill cancer cells of the NCI-H1688 lineage more than non-tumor cells (MRC-5). However, the isolated myristicin showed a low toxicity to the cell lines [42].

In addition to the products mentioned, a study carried out tests on the antiproliferative activity of essential oils obtained from flowering aerial parts (containing 16.5% of myristicin) and ripe fruits (containing 15.3% of myristicin) of the *Echinophora spinosa* plant. Both oils tested were toxic to U937 cells, but the fruit oil was much more cytotoxic. Although myristicin may have contributed to the cytotoxicity of the oils, the difference between the results was attributed to other components [43].

Through these data, it is not possible to conclusively establish the antiproliferative activity of myristicin. Although some of the studies presented have shown that it is capable of inducing cellular mechanisms that lead to apoptosis (Figure 2), other articles have shown that it was not able to reduce cell viability in some cell lines. Therefore, further studies are needed to prove its effectiveness, covering several cell lines, and carrying out more detailed studies to elucidate the mechanisms of action of the substance. Above all, it is important that further research is carried out with isolated or purified myristicin, to eliminate interference from other compounds present in the analyzed plant extracts and essential oils.

### 2.5. Antimicrobial Activity

The antimicrobial activity of myristicin has been widely studied in the last decade, but there are still divergences regarding its in vitro effects and mechanisms of action.

Among the substances investigated, the essential oils of *Myristica fragrans* (nutmeg), *Heracleum transcaucasicum*, *Heracleum anisactis*, *Anethum graveolens* (dill), *Apium nodiflorum*, *Petroselinum crispum* (parsley), *Pycnocycla bashagardiana* and *Piper sarmentosum*, all containing high concentrations of myristicin, ranging between 12% and 96% of the composition, are noteworthy. In addition, crude extracts of Athamanta sicula and isolated myristicin with a high degree of purity were tested. The inhibition of growth promoted by these substances was evaluated by means of disk diffusion assays, microdilution, determination of the minimum inhibitory concentration (MIC) and in silico assays. Different species of bacteria and fungi were tested [8,22,35,44,45,46,47,48,49,50,51,52].

Some studies showed that the essential oils of *Heracleum transcaucasicum* and *Heracleum anisactis* (containing 96.87% and 95.15% of myristicin, respectively), the *Athamanta sicula* plant extract, as well as the myristicin isolated from the plant, showed weak or absent activity against the species tested: *Staphylococcus aureus*, *Staphylococcus epidermidis*, *Escherichia coli*, *Pseudomonas aeruginosa*, *Candida albicans* and *Candida tropicalis*. In a study that tested the essential oil of nutmeg with different concentrations of myristicin, it was found that those with higher amounts (ranging from 26% to 38%) had no inhibitory effect against *Escherichia coli, Aspergillus fumigatus*, and methicillin-resistant *Staphylococcus aureus* (MRSA), *Pseudomonas aeruginosa*, *Klebsiella pneumoniae*, and were slightly active against *Cryptococcus neoformans* [8,22,35,44].

In a study carried out to evaluate the fungicidal activity on several species, essential oils and *Apium nodiflorum* extracts containing 29% of myristicin were tested. The results showed a variability of inhibition among all strains of fungi tested, being especially active against dermatophytes. Moreover, for *Cryptococcus neoformans*, there was significant activity. For *Aspergillus* spp., the oil proved to be less effective. However, this activity was attributed to a synergistic effect between myristicin and dilapiol, another substance present in the plant [46].

Other studies showed that the essential oil of nutmeg (*Myristica fragrans*) containing only 10% of myristicin was able to strongly inhibit the growth of the fungi *Aspergillus flavus* and *Aspergillus ochraceus*. The essential oil of the *Pycnocycla bashagardiana* plant containing 39% myristicin exhibited strong antimicrobial activity against *Staphylococcus aureus, Staphylococcus epidermidis, Escherichia coli* and *Candida albicans*. Essential oils of dill (*Anethum graveolens*) and parsley (*Petroselinum crispum*), containing from 28% to 42% of myristicin, were able to inhibit the following microorganisms: *Escherichia coli*, *Staphylococcus albus*, *Bacillus mesentericus* and *Aspergillus flavus*. The essential oil of parsley (*Petroselinum crispum*) containing 14% of myristicin showed fungistatic and fungicidal activity against *Aspergillus fumigatus*, *Aspergillus niger*, *Aspergillus ochraceus*, *Aspergillus versicolor*, *Penicillium funiculosum*, *Penicillium ochrochloron*, *Penicillium verrucosum* and *Trichoderma viride*, and inhibited the growth of bacteria *Bacillus cereus, Enterobacter cloacae, Escherichia coli, Listeria monocytogenes*, *Pseudomonas aeruginosa*, *Salmonella enterica* and *Staphylococcus aureus* with varying degrees of sensitivity. A study that aimed to investigate the activity of myristicin in combating acne tested the extract and essential oil of nutmeg (*Myristica fragrans*) against the bacteria *Cutibacterium acnes* and *Staphylococcus aureus*, and presented a good antibacterial effect against both [26,46,47,48,49].

Myristicin isolated from the essential oil of *Piper sarmentosum* (representing about 81% to 83% of its composition) was able to inhibit the proliferation of *Escherichia coli* in vitro. The study that demonstrated this activity also revealed that myristicin was able to inhibit, in vitro, the activity of the GTPase enzyme, interfering with a fundamental step for cell division [50].

A computer assay performed with myristicin tested its ability to inhibit the multi-drug resistant bacterial strains growth: *Bacillus anthracis*, *Escherichia coli*, *Staphylococcus aureus*, *Streptococcus pneumoniae* and *Mycobacterium tuberculosis*. The results obtained showed that myristicin would be effective against *Streptococcus pneumoniae*, as it would be able to inhibit the bacterial folic acid biosynthesis dihydropteroate synthase enzyme (DHPS) [51].

Myristicin was also evaluated for its ability to protect food against aflatoxins produced by certain fungi. In this study, the essential oil of nutmeg containing 21% of myristicin was used, which was able to inhibit the growth of the strain of *Aspergillus flavus* that produced the most aflatoxin in vitro. Furthermore, it was shown that the oil caused a decrease in the ergosterol content of the fungus’s plasma membrane, which caused cellular ion leakage [52].

After surveying these data, it is possible to conclude that myristicin may have selective antimicrobial activity on some species (Table 1, Figure 2). However, many of the results (positive or negative for antimicrobial activity) observed in the studies can be attributed to the interaction between myristicin and other compounds, as they can either potentiate or inhibit its effect. Therefore, it is necessary to carry out further studies with the isolated molecule to assess its antiproliferative potential against each species, as well as elucidating the mechanisms by which it can inhibit growth or destroy microbial cells.

### 2.6. Insecticide and Larvicide Activity

The insecticidal and larvicide activities of myristicin were the properties described in recent years that showed the most satisfactory results in vivo and in vitro. The materials used in the research were essential oils extracted from plants such as *Piper aduncum*, *Echinophora spinosa*, *Clausena anisum-olens*, *Helosciadium nodiflorum*, *Ligusticum pteridophyllum*, *Trachyspermum ammi*, *Smyrnium olusatrum*, *Pimpinella anisum*, *Myristica fragrans* (nutmeg), *Ligusticum jeholense* or isolated myristicin [53,54,55,56,57,58,59,60].

In general, myristicin had a potentially toxic effect on the studied insect species: Liposcelis bostrychophila and Lasioderma serricorne, *Culex pipiens* (larva), *Aedes aegypti, Euschistus heros*, *Culex quinquefasciatus* (larva), *Spodoptera littoralis* (larva), *Musca domestica* (adult), and *Spodoptera littoralis*, *Trichoplusia ni*, *Tribolium castaneum*, *Lasioderma serricorne*, *Liposcelis bostrychophila* and *Microcerotermes beesoni*. Several researches have shown the inhibition of the enzyme acetylcholinesterase and cytochrome detoxifying enzymes. This inhibition blocks the transmission of nerve impulses in the insect, causing paralysis and death [53,56,57,58,59,60].

The essential oil obtained from the plant *Clausena anisum-olens* and the myristicin isolated from this oil showed contact toxicity against both adult insects species *Liposcelis bostrychophila* and *Lasioderma serricorne*. The isolated myristicin showed 92% repellency against *Liposcelis bostrychophila* and 68% repellency against *Lasioderma serricorne*, while the plant’s essential oil showed only 32% to 38% of its toxic effect [53]. 

The essential oil of the *Piper aduncum plant*, which has 30% myristicin, showed histopathological toxicity against the insect *Euschistus heros*, with cytological changes and tissue ruptures, characterizing an increase in mitochondria population and a loss of glycogen and lipids. The salivary glands, as well as the midgut, are affected by the oil, showing an insecticidal activity [56].

The insecticidal property of *Echinophora spinosa* roots’ and leaves’ essential oils containing 47% and 2.7% of myristicin, respectively, were evaluated against larvae of the species *Culex quinquefasciatus*, *Spodoptera littoralis* and adult insects of *Musca domestica*. Insects were subjected to various concentrations of the oils to determine the LC50 (50% lethal concentration). The results show a greater efficacy of the root, as the LC50 was lower, indicating that myristicin may be the substance responsible for the effect [57]. Another study was conducted with the same insect species and used *Helosciadium nodiflorum* as a source of myristicin. In this case, the plant contained 35% myristicin, and was subjected to the hydrodistillation process, obtaining an essential oil that was then tested on insects. The result showed that myristicin has a toxic effect on insects, and its mechanism was attributed to synergistic effects between myristicin and other components [58].

Essential oils of plant species *Helosciadium nodiflorum* collected from different localities (containing 49% and 24% of myristicin), as well as the isolated substance, were also investigated for insecticidal activity against *Trichoplusia ni*. The essential oil had a stronger toxic effect than myristicin alone; however, among the compounds tested in isolation, myristicin was the most potent. Its toxicity is a consequence of inhibition of the CYP450 enzyme in insects [61].

Adult insects of *Tribolium castaneum*, *Lasioderma serricone* and *Liposcelis bostrychophila* were exposed to essential oil containing 90% myristicin, fluid extract containing 48% of it, as well as myristicin isolated from the *Ligusticum pteridophyllum* plant. All tested components showed insecticidal activity, but myristicin alone exhibited a more potent action as the LD50 value was lower [62].

The insecticidal activity of the essential oil of nutmeg containing 6% myristicin was evaluated against termite *Microcerotermes beesoni*. The results showed that the LC50 value of the essential fruit oil is 28.6 mg. Treatment for 14 days with 5 mg of myristicin resulted in 100% mortality [50]. The myristicin found in the roots and rhizomes of *Ligusticum jeholense* showed contact toxicity and repellency. When in contact with *Tribolium castaneum* and *Lesiodwema serricorne*, myristicin exerted its insecticidal and repellent effect on target insects [51].

Pure myristicin, *Peperomia borbonensis* essential oil (containing 39% of the substance) and a mixture of myristicin and elemicin (which are the main components of the oil) insecticidal activity was evaluated against *Bactrocera cucurbitae* insects. The oil showed a neurotoxic effect as a consequence. Soon after contact with the association of myristicin and elemicin, the flies had convulsions and were knocked down. Isolated myristicin has led to only 40% mortality. Thus, it is noted that myristicin has insecticide properties for the studied species, but it is enhanced when there is the presence of other components of the plant’s essential oil [63].

Studies conducted with essential oils (containing 20.39% myristicin) and isolated myristicin obtained from *IIIicium henryi* root bark revealed insecticidal activity against *Liposcelis bostrychophila* lice. The oils and isolated myristicin showed strong contact and fumigant toxicity for insects and myristicin was the most potent compound [64].

Essential oils from plants of the *Apiaceae* family, with a 99% myristicin presence, were examined as larvicides for the Asian tiger mosquito species (*Aedes albopictus*). The research showed a 95% mortality result for mosquito larvae treated with a concentration of 0.1 mg/mL of oil [65].

In a research to evaluate the larvicidal activity against *Culex quinquefasciatus* larvae, essential oils from *Sison amomum* and *Echinophora spinosa* (with 41% myristicin) were used, as well as isolated myristicin, and also oils that did not contain myristicin obtained from *Heracleum sphondylium*, *Heracleum sphondylium subsp. ternatum* and *Trachyspemum ammi*. The study showed that among all the oils tested, the second most toxic was the one containing myristicin, and isolated myristicin also has a potential for larvicidal capacity [66]. 

An in vivo study, which evidenced the larvicidal activity of myristin against *Culex pipiens* larvae, reports that myristicin had a potent toxic activity for the larvae. The test to verify the insecticidal effects of myristicin isolated from nutmeg essential oil against *Culex pipiens* and *Aedes aegypti* insects were also carried out. The study performed was a vapor toxicity test in adult mosquitoes. Myristicin had a more potent larvicidal capacity than oil against the investigated insect. The *Culex pipiens* mosquito is more susceptible to the activity of both compared to *Aedes aegypti* [54,55].

According to the data presented, we conclude that myristicin is a natural substance, with insecticidal and larvicide capacity being an alternative to chemical products that are also used for the same purposes.

### 2.7. Other Activities

**Figure 3:**
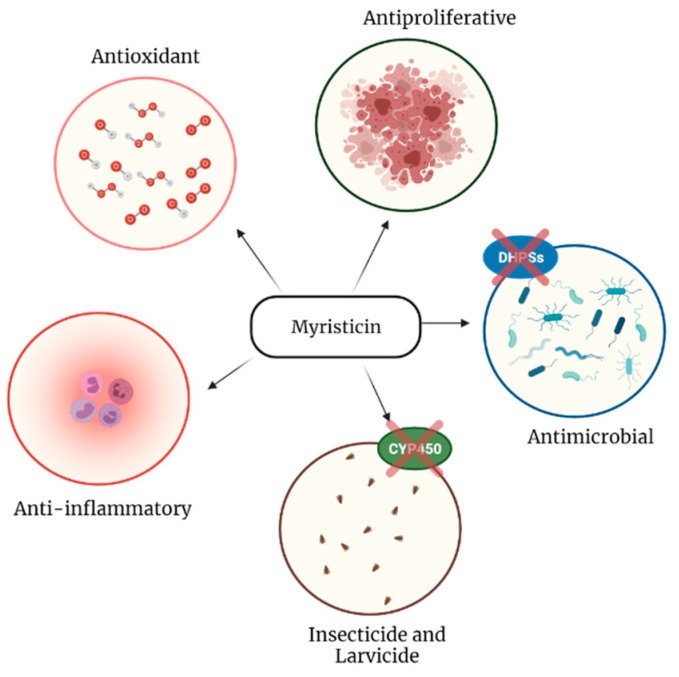
Graphical representation of the main biological activities of myristicin.

Figure 3 and Table 1 summarizes the main biological acitivities of myristicin and its mechanism of action studied until now. There are published studies on other biological activities of myristicin, but little is reported in the literature. However, they point out promising paths for new therapeutic properties, and that is why it is relevant to continue studying them.

A publication on the aqueous extract of the aerial part of parsley (*Petroselinum crispum*) sought to investigate the antihypertensive activity of the plant. In vivo studies were performed with male albino rats, and an in vitro study used isolated thoracic aorta rings. The results show a potent vasorelaxant activity in aortic vascular rings, while in animals the extract induced a decrease in blood pressure parameters. More detailed studies showed that there was a blockage of calcium channels present in the vascular wall, but also suggest that other pathways may be involved in the antihypertensive effect such as, for example, increased nitric oxide synthesis [67].

The ability of myristicin to protect neurons from hypoxia-induced injuries was investigated. To conduct these assays, rat dorsal root ganglion (DRG) neurons were used. The results showed that myristicin reduced the viability of neurons when exposed to concentrations greater than 50 mM. However, at lower concentrations, it significantly increased cell viability in neurons when exposed to hypoxia, as it protected against hypoxic injury, not causing apoptosis. Complementary trials showed that myristicin decreased cleaved caspase-3 and bcl-2 levels in these hypoxia-induced neurons. Therefore, it was observed that it can reverse hypoxia-induced apoptosis in DRG neurons, affecting protein expression levels of molecules associated with apoptosis. Furthermore, myristicin decreased the malondialdehyde (MDA) content and the release of lactate dehydrogenase (LDH) enzymes, and positively regulates the activities of superoxide dismutase (SOD) and glutathione peroxidase (GSH-PX). These enzymes are involved in the hypoxia process, and this modulation may have been responsible for the protective effect of myristicin [68].

A study conducted with myristicin isolated from the leaves of *Perilla frutescens* sought to investigate whether it induces MUC5AC gene expression and mucin production by airway epithelial cells. Tests were then performed with NCI-H292 cells (mucoepidermoid lung carcinoma), and the results showed that myristicin significantly inhibited gene expression and production of MUC5AC from NCI-H292 cells. Furthermore, it suppressed the production of mucin protein MUC5AC induced byepidermal growth factor (EGF), although it did not affect TNF-α-induced mucin protein MUC5AC production [69].

The pro-sexual effects of myristicin were also investigated. A study conducted with an aqueous extract of *Piper auritum* on Wistar rats showed that, in males with delayed ejaculation, the extract stimulated ejaculatory behavior and recovered the electromyographic activity of the pelvic muscles, participating in seminal emission and ejaculation. The most relevant action provoked by the PA was to increase the number of GMPEs (genital motor ejaculation pattern), thus restoring the ejaculatory capacity. The pro-sexual effects of *Piper auritum* produced on ejaculatory function are related to the participation of several neurotransmitter systems, and with a prominent role of 5-HT1A serotonin receptors. Although several other compounds are present in the extract, myristicin has a pro-serotoninergic action, indicating that it may be responsible for the action shown in the study [13,70].

An interesting publication discusses the anticonvulsant and inhibitory effects on glial activation of *Myristica fragrans* (nutmeg) extract. This material, containing about 11% myristicin, was tested in male NMRI rats that were induced to have seizures. Behavioral studies have shown that pretreatment with nutmeg extract effectively reduced seizure behavior, decreased cell death in the hypothalamus and improved glial activation [71].

Still regarding the actions of myristicin on the central nervous system, a study on the antidepressant potential of *Myristica fragrans* was published. Male Wistar rats were submitted to tests for analysis of antidepressant activity, using imipramine as a control and nutmeg extract as a test. Myristicin was not quantified in this extract; however, as it is the major chemical component of seeds, it is possible to correlate its presence with the results obtained. The extract exhibited an action very similar to the group treated with imipramine, demonstrating the potential antidepressant activity of the species [72].

Interestingly, the effects of myristicin on appetite were assessed through inhalation. In this study, male ddY mice inhaled essential oil of nutmeg and myristicin isolated from the oil. In both cases, there was an increase in appetite and weight gain in mice. However, this effect was lost when the compounds were administered daily to mice (after 8 days) [73].

Another study sought to assess the anti-obesity effect of nutmeg. The essential oil of *Myristica fragrans* showed a high binding activity to the CB1 cannabinoid receptor. Blocking this receptor reduces appetite and stimulates lipid metabolism, which would cause the anti-obesity effect. However, the author relates this activity to a synergism between myristicin and other compounds found in nutmeg essential oil [74].

The hepatocarcinogenic effects of myristicin were also evaluated by a study mentioned by Chen et al. (2016). For this, medium white turkey eggs with 22- to 24-day-old fetuses received myristicin injections (25.50 mg/egg), and measurement of DNA strand breaks in fetal livers was performed. The 50 mg/egg dose induced a significant increase in DNA support breaks, in addition to reducing cell viability by 50%. This result indicates a genotoxic and carcinogenic potential [75].

A study published in 2013 sought to assess the molluscicidal activity of myristicin. To evaluate this effect, the snail vector *Lymnaea acuminata* was exposed to isolated myristicin, and to combinations of nutmeg and myristicin with piperonyl butoxide (PB) as a synergist. Both myristicin alone and nutmeg powder treatment were more potent when administered together with PB. Noteworthy, myristicin alone also showed molluscicidal activity [76].

These studies, despite dealing with little-explored activities of myristicin, demonstrate how much of this molecule’s potential can still be explored through further research in various applications.

### 2.8. Future Perspectives

Myristicin is a molecule that is still poorly studied and is still not used in therapy, but the few available data point to a promising therapeutic potential. Considering the need of the pharmaceutical industry worldwide to obtain new treatments for diseases such as cancer and infections, it becomes relevant to clinically evaluate the use of substances that have shown therapeutic potential in preliminary studies. We encourage researchers to explore this promising molecule and carry out more detailed studies about its mechanism of action, especially on its anti-inflammatory, antiproliferative and antioxidant activites.

## 3. Materials and Methods

The study was carried out through a literature review. Pubmed, Lilacs and Scielo platforms were used to search for articles from the last 10 years, using the keywords myristicin and therapeutic properties. Through these platforms, 94 articles were found that conducted biological activity assays involving myristicin. However, only those in which the amount of myristicin in the sample was relevant were selected. A total of 68 references were included in this review. 

## 4. Conclusions

Myristicin is an alkylbenzene present in the roots, fruits and aerial parts of several plant species used for centuries by countless populations, both as food and as natural medicine treatments. After this survey of literature data, it is evident that this is a promising molecule, with several properties, such as antimicrobial, insecticide, larvicide, psychoactive and therapeutics, among which the following stand out: antioxidant, anti-inflammatory and antiproliferative. These data demonstrate myristicin is a great potential to be explored in medicine to be used as a clinical treatment for pathologies, justifying the conduct of new studies focusing on its mechanism of action.

## Figures and Tables

**Table 1:** The main biological activities of myristicin and its known mechanisms.

Biological Activity	Mechanisms	Species/Cell Lines
Antioxidant	Increases the concentration of catalase, superoxide dismutase, glutathione peroxidase glutathione reductase and decreases levels of lipid peroxidation	-
Anti-inflammatory	Inhibits PGE2, COX-2, tumor necrosis factor alpha (TNF-a), interleukins (IL-1, IL-6, IL-8, IL-10 and IL-17), nitric oxide (NO), macrophage inflammatory proteins (MIP-1α r MIP 1β), colony stimulating factor (GM-CSF), IP-10, MCP-1, MCP-3 and myeloperoxidase (MPO)	RAW 264.7, A549, HEK293, HL-60 and human fibroblast cells
Antiproliferative	Induces cell apoptosis through changes in mitochondrial membrane potential, cytochrome C release, caspase-3 activation, PARP cleavage, fragmentation of DNA, down-regulation of DNA damage response genes and reduces the expression of bcl-2 gene	K-562, NCI-H460, MCF-7, KB cell line, RD cells, Caco-2, AA8 and EM9, HepG2, NCI-H1688, MRC-5, U937
Antimicrobial	Inhibition of the polymerization of FtsZ, of the enzyme dihydropteroate synthases (DHPSs) and of the GTPase enzyme	Fungi: *Aspergillus flavus*, *Aspergillus fumigatus*, *Aspergillus niger*, *Aspergillus ochraceus*, *Aspergillus versicolor*, *Penicillium funiculosum*, *Penicillium ochrochloron*, *Penicillium verrucosum*, *Trichoderma viride* Bacteria: *Bacillus cereus*, *Enterobacter cloacae*, *Escherichia coli*, *Listeria monocytogenes*, *Pseudomonas aeruginosa*, *Salmonella enterica*, *Staphylococcus aureus*, *Cutibacterium acnes*
Insecticide and larvicide	Inhibition of the CYP450 enzyme and acetylcholinesterase in insects	*Liposcelis bostrychophila* and *Lasioderma serricorne*, *Culex pipiens* (larva), *Aedes aegypti*, *Euschistus heros*, *Culex quinquefasciatus* (larva), *Spodoptera littoralis* (larva), *Musca domestica* (adult), and *Spodoptera littoralis, Trichoplusia ni*, *Tribolium castaneum*, *Lasioderma serricorne*, *Liposcelis bostrychophila* and *Microcerotermes beesoni*

## Data Availability

Not applicable.
